# The hemoglobin, albumin, lymphocyte, and platelet score as a useful predictor for mortality in older patients with hip fracture

**DOI:** 10.3389/fmed.2025.1450818

**Published:** 2025-02-18

**Authors:** Zhicong Wang, Hailong Liu, Mozhen Liu

**Affiliations:** ^1^Department of Orthopedics, The First Affiliated Hospital of Dalian Medical University, Dalian, Liaoning, China; ^2^Department of Orthopedics, People’s Hospital of Deyang City, Deyang, Sichuan, China

**Keywords:** HALP score, mortality, hip fracture, older adults, predictor, biomarker

## Abstract

**Objective:**

With a rapidly aging population, identifying effective prognostic predictors has always been a research hotspot in older patients with hip fracture. Recently, the score combining hemoglobin, albumin, lymphocyte and platelet (HALP) has been proposed as a novel biomarker for reflecting systemic inflammation and nutritional status. However, it is unclear whether the HALP score could be a potential prognostic indicator for mortality following hip fracture. Thus, the objective of this study was to explore the relationship between the HALP score and mortality risk in older patients with hip fracture, and further evaluate its predictive value.

**Methods:**

Consecutive patients were identified from our institutional hip fracture database over the period from January 1, 2013 to December 31, 2022. Outcomes were 90-day all-cause mortality and overall mortality at the longest follow-up. Multivariate Cox proportional hazard models and restricted cubic spline (RCS) models were employed to assess this relationship. The incremental predictive performance of the HALP score was assessed using the c statistic, net reclassification improvement (NRI) and integrated discrimination improvement (IDI).

**Results:**

In total, 1707 patients were included with a median follow-up duration of 44.4 months, of whom 174 cases (10.2%) died within 90 days and 667 cases (39.1%) died at the longest follow-up. After multivariate adjustment, compared to tertile 1 group, tertile 2 and tertile 3 groups exhibited 0.676-fold (95% confidence interval [*CI*]: 0.471–0.972, *p* = 0.035) and 0.598-fold (95% *CI*: 0.390–0.918, *p* = 0.019) lower risks of 90-day mortality, as well as 0.681-fold (95% *CI*: 0.566–0.820, *p* < 0.001) and 0.618-fold (95% *CI*: 0.504–0.759, *p* < 0.001) decreased risks of overall mortality, respectively. For each unit increase in the HALP score, there was a significant decrease in 90-day mortality by 1.1% and in overall mortality by 1.0% (all *p* < 0.05). RCS analyses indicated nonlinear relationships between the HALP score and 90-day and overall mortality (all *P* for nonlinear <0.01). Moreover, adding the HALP score significantly improved the predictive ability for 90-day and overall mortality (all *p* < 0.05).

**Conclusion:**

The HALP score was independently associated with mortality risk, highlighting its potential as a useful prognostic indicator for predicting mortality in older patients with hip fracture.

## 1 Introduction

With the acceleration of the aging process and the increase of the older population, hip fractures have emerged as a worldwide public health challenge, characterized by high incidence rates, substantial mortality, considerable disability, and considerable healthcare expenditures (1–6). Based on data from the Global Burden of Disease (GBD) Study 2019, the incidence of hip fractures was estimated at 14.2 million cases, with an associated burden of approximately 2.9 million years lived with disability (5, 7). Besides this, an international study involving 19 countries and regions reported a median 1-year all-cause mortality rate of 22.4% following hip fracture (4). As is well known, timely identification of hip fracture patients who are at elevated risk of death is essential for facilitating prompt interventions and guiding personalized treatment strategies, thereby improving the prognosis (8–12). Therefore, the search for useful prognostic predictors has always been a focus of research in hip fracture patients (13–15).

Considering their accessibility and affordability in daily clinical practice, blood indicators have attracted increased attention as biomarkers of mortality among hip fracture patients, such as hemoglobin (16, 17), lymphocyte (18), platelet (19), and albumin (17, 18). In addition, the prognostic nutritional index (PNI), derived from albumin concentration and lymphocyte count, has been shown to independently predict unfavorable survival outcomes in hip fracture patients at 2 years of follow-up (20). In our previous study, an elevated platelet-to-lymphocyte ratio (PLR) was correlated with higher 1-year mortality rate in older patients with hip fracture (21). More recently, the score combining hemoglobin, albumin, lymphocyte and platelet (HALP) has been proposed as a promising novel biomarker for reflecting systemic inflammation and nutritional status simultaneously (22, 23). Emerging studies have highlighted the potential utility of the HALP score in predicting mortality in several diseases, including cancer (e.g., hepatocellular carcinoma, breast cancer) (24, 25), cardiovascular disease (e.g., myocardial infarction, heart failure, coronary heart disease) (26–28), respiratory disease (e.g., chronic obstructive pulmonary disease) (29), and even in the general population (23). Given the clear associations between hemoglobin, lymphocyte, platelet, albumin and survival (16–19), we hypothesized that the HALP score may serve as an independent prognostic indicator for mortality following hip fracture.

As far as we know, no prior study has assessed the association between the HALP score and mortality in hip fracture patients who have a high risk of death. Therefore, the first aim was to investigate this potential association in older patients with hip fracture. Moreover, little is known about the predictive ability of the HALP score for mortality, which may limit its further clinical application. Consequently, the second objective was to explore its incremental predictive value.

## 2 Materials and methods

### 2.1 Patients and study design

This retrospective observational cohort study was conducted using our institutional hip fracture database, which has been widely utilized for prognostic research (21, 30–34). Inclusion criteria for the database was a diagnosis of hip fracture at People’s Hospital of Deyang City, which was radiographically confirmed by imaging examinations. Patients were excluded based on the following criteria: (1) age under 60 years; (2) high-energy fractures caused by traffic accidents or falls from a height, old fractures more than three weeks after the injury, pathological fractures due to tumor or infection, periprosthetic fractures following joint replacement; (3) absence of any follow-up information. Between January 1, 2013 and December 31, 2022, a total of 1721 patients with hip fracture were recorded in this database. In this study, patients with missing laboratory data on hemoglobin, lymphocyte, platelet or albumin were further excluded. The study flow is illustrated in Supplementary Figure S1. The study protocol was reviewed and approved by the Hospital Ethics Committee (No. 2021–04-091-K01). This study was conducted in adherence to the principles of the Declaration of Helsinki, and all participants provided written informed consent for the utilization of their clinical data for research purposes.

### 2.2 Data collection

The methods for data collection have been reported previously (34). The following variables were retrieved from the database: (1) demographic information included age (years), sex (male or female), height (m), weight (kg), marital status [widowed or other (including single, married or divorced)], and smoking [no or yes (including current and former smoking)]. Body mass index (BMI) is computed using the formula: weight (kg) divided by height squared (m^2^). (2) Patient comorbidities were quantified using the Charlson Comorbidity Index (CCI). The CCI was derived from 17 specified diseases and categorized into three levels: none (CCI = 0), low (CCI = 1), and high (CCI ≥ 2) (35). (3) Fracture type included femoral neck fracture and intertrochanteric fracture. (4) First laboratory data after admission included hemoglobin, lymphocyte, platelet and albumin. The HALP score was computed using the formula: hemoglobin (g/L) × albumin (g/L) × lymphocyte (/L) / platelet (/L), and participants were divided into three groups based on the HALP tertiles (23). In addition, several laboratory indicators associated with the mortality risk of hip fracture were also collected, including neutrophil (36), monocyte (36), creatinine (36), glucose (19), internationalization standardization rate (INR) (36), calcium (19, 37), sodium (17), and potassium (18).

### 2.3 Follow-up and mortality

The outcomes of this study were all-cause death within 90 days of admission (90-day mortality) and at the longest follow-up (overall mortality). As described in our previous study (34), patients’ survival information were retrieved from their medical records, including death certificates and the latest clinical records after discharge (outpatient consultations, emergency department visits, and any readmissions), or telephone interviews (last follow-up date: April, 2023). If the survival status could not be determined using the aforementioned approaches, participants were classified as lost to follow-up. In this database, the rate of loss to follow-up was 5.80% (106 out of 1827). Survival time was calculated as the interval from hospital admission to either death or the last follow-up, whichever came first.

### 2.4 Statistical analysis

Prior to analyses, all variables were examined for missing values. The numbers of missing data were as follows: height (*n* = 222, 13.0%), weight (*n* = 190, 11.1%), creatinine (*n* = 3, 0.2%), glucose (*n* = 12, 0.8%), INR (*n* = 32, 1.9%), calcium (*n* = 12, 0.7%), sodium (*n* = 12, 0.7%) and potassium (*n* = 13, 0.8%). These missing values were imputed using a random forest-based multiple imputation by chained equations (MICE) under the assumption of missing at random (38). Density plots showed the distribution of the missing data before and after imputation, indicating adequate imputation (Supplementary Figure S2). After imputation, continuous variables are described as mean ± standard deviation (SD) or median [first quartile (Q1), third quartile (Q3)] according to the results of Shapiro–Wilk test for normality, and comparisons among groups were performed using one-way analysis of variance (ANOVA) or Kruskal-Wallis rank-sum test. Categorical variables are reported as number (percentage) and were compared using the chi-square test. Additionally, violin plots were constructed to visually compare the HALP score distributions between dead and alive patients, and differences were assessed by Wilcoxon rank-sum test.

For time-to-event analysis, Kaplan–Meier survival curves were constructed and compared using the log-rank test. The median follow-up duration was determined using the reverse Kaplan–Meier approach. Univariate Cox proportional hazards analyses were initially conducted to identify potential prognostic factors associated with survival (Supplementary Table S1). Subsequently, factors with a *p* value <0.2 were selected for inclusion in the multivariate Cox regression model. Hazard ratios (HR) and their corresponding 95% confidence intervals (*CI*) were calculated to estimate the risk of death. Specifically, multivariate Cox regressions were adjusted for demographic variables (age, sex, BMI, marital status, smoking), comorbidity (CCI), fracture type, and laboratory data (neutrophil, monocyte, creatinine, glucose, INR, calcium, sodium, potassium). In these models, the HALP score was entered as a continuous variable (per unit increase) and as a categorical variable (the lowest tertile as the reference). To test the linear trend across categories, the median values of each HALP tertile was assigned and analyzed as a continuous variable (tertile 1: 13.39, tertile 2: 26.81, tertile 3: 46.47). The proportional hazard assumption was tested with Schoenfeld residuals, and no violation was found. We checked multicollinearity among the included variables using the variance inflation factor (VIF), considering VIF ≥ 5 as indicative of significant multicollinearity (39). No evidence of multicollinearity was detected in any of the models (Supplementary Table S2).

In addition, we explored the relationship within subgroups categorized by age (< 80.0 and ≥ 80.0 years), sex (male and female), BMI [underweight (< 18.5 kg/m^2^), normal weight (18.5–24.9 kg/m^2^), and overweight (≥ 25.0 kg/m^2^)], marital status (widowed and other), CCI (none, low and high), and fracture type (femoral neck fracture and intertrochanteric fracture). Potential interactions between the stratification factors and HALP score were tested. A sensitivity analysis using complete data was conducted to evaluate the potential influence of missing data on the study results. Restricted cubic spline (RCS) models were employed to investigate the potential nonlinear associations between the HALP score and mortality risk. The optimal number of knots was determined based on the minimum Akaike information criterion (AIC), considering a range from 3 to 7 knots. As shown in Supplementary Table S3, RCS models with three knots were selected. To assess the incremental predictive performance of the HALP score compared to the established CCI model, we calculated the c statistic, continuous net reclassification improvement (NRI) and integrated discrimination improvement (IDI) (40).

All reported *p* values are two-sided, with *p* < 0.05 indicating statistical significance. Statistical analyses were conducted using R statistical software (version 4.4.0; R Project for Statistical Computing).

## 3 Results

### 3.1 Patient characteristics

The final analysis included 1707 patients, and their baseline characteristics are summarized in Table 1. The median age of patients was 80.0 years, 35.5% were males, and the distribution of comorbidities was as follows: 45.6% had no comorbidities, 30.4% had low comorbidity, and 24.0% had high comorbidity. Intertrochanteric fracture was the most common fracture type (51.4%), followed by femoral neck fracture (48.6%). The first and second tertile cutoff values of the HALP score were 20.14 and 34.82, and patients were grouped into tertile 1 (≤ 20.14, *n* = 569), tertile 2 (20.15–34.82, *n* = 569), and tertile 3 (≥ 34.83, *n* = 569). When these groups were compared, statistically significant differences were observed in age, BMI, marital status, CCI, fracture type, neutrophil, creatinine, glucose, INR, calcium and sodium (all *p* < 0.05).

**Table 1:** Patient characteristics stratified by tertiles of the HALP score.

Characteristics	Total (*n* = 1707)	Tertile 1 (≤ 20.14, *n* = 569)	Tertile 2 (20.15–34.82, *n* = 569)	Tertile 3 (≥ 34.83, *n* = 569)	*p* value
Demographics					
Age, years, median (Q1, Q3)	80.0 (73.0, 85.0)	82.0 (77.0, 87.0)	79.0 (73.0, 85.0)	78.0 (70.0, 83.0)	<0.001
Sex, male, *n* (%)	606 (35.5)	205 (36.0)	204 (35.9)	197 (34.6)	0.864
BMI, kg/m^2^, median (Q1, Q3)	21.2 (19.1, 23.9)	20.0 (18.4, 22.5)	21.5 (19.3, 24.3)	22.2 (20.0, 24.6)	<0.001
Marital status, widowed, *n* (%)	449 (26.3)	170 (29.9)	151 (26.5)	128 (22.5)	0.018
Smoking, *n* (%)	396 (23.2)	123 (21.6)	142 (25.0)	131 (23.0)	0.408
CCI, *n* (%)					<0.001
None	778 (45.6)	201 (35.3)	277 (48.7)	300 (52.7)	
Low	519 (30.4)	183 (32.2)	172 (30.2)	164 (28.8)	
High	410 (24.0)	185 (32.5)	120 (21.1)	105 (18.5)	
Fracture type, *n* (%)					<0.001
Femoral neck fracture	829 (48.6)	214 (37.6)	280 (49.2)	335 (58.9)	
Intertrochanteric fracture	878 (51.4)	355 (62.4)	289 (50.8)	234 (41.1)	
Laboratory findings					
Neutrophil, ×10^9^/L, median (Q1, Q3)	7.0 (5.1, 9.4)	7.2 (5.2, 10.0)	7.3 (5.3, 9.5)	6.5 (4.8, 8.7)	< 0.001
Monocyte, ×10^9^/L, median (Q1, Q3)	0.52 (0.39, 0.67)	0.50 (0.38, 0.67)	0.52 (0.39, 0.66)	0.53 (0.40, 0.67)	0.287
Creatinine, μmol, median (Q1, Q3)	66.4 (55, 86)	68.0 (55.0, 96.0)	68.0 (55.4, 87.0)	63.0 (54.0, 79.0)	<0.001
Glucose, mmol/L, median (Q1, Q3)	6.9 (5.7, 8.8)	7.2 (6.0, 9.1)	7.0 (5.8, 8.9)	6.4 (5.5, 8.3)	<0.001
INR, median (Q1, Q3)	1.05 (1.00, 1.12)	1.06 (1.01, 1.13)	1.05 (1.00, 1.12)	1.04 (0.99, 1.11)	<0.001
Calcium, mmol/L, mean ± SD	2.15 ± 0.17	2.12 ± 0.18	2.16 ± 0.16	2.17 ± 0.16	<0.001
Sodium, mmol/L, median (Q1, Q3)	141.5 (139.1, 143.7)	140.7 (138.1, 143.2)	141.6 (139.1, 143.7)	142.0 (139.9, 144)	<0.001
Potassium, mmol/L, median (Q1, Q3)	3.90 (3.60, 4.22)	3.94 (3.59, 4.27)	3.90 (3.63, 4.23)	3.89 (3.58, 4.16)	0.137

Q1, 25th percentile; Q3, 75th percentile; *n*, number; BMI, body mass index; CCI, Charlson Comorbidity Index; INR, international normalized ratio. The *p* value refers to the comparison among tertiles.

### 3.2 Association between the HALP score and mortality

**Figure 1:**
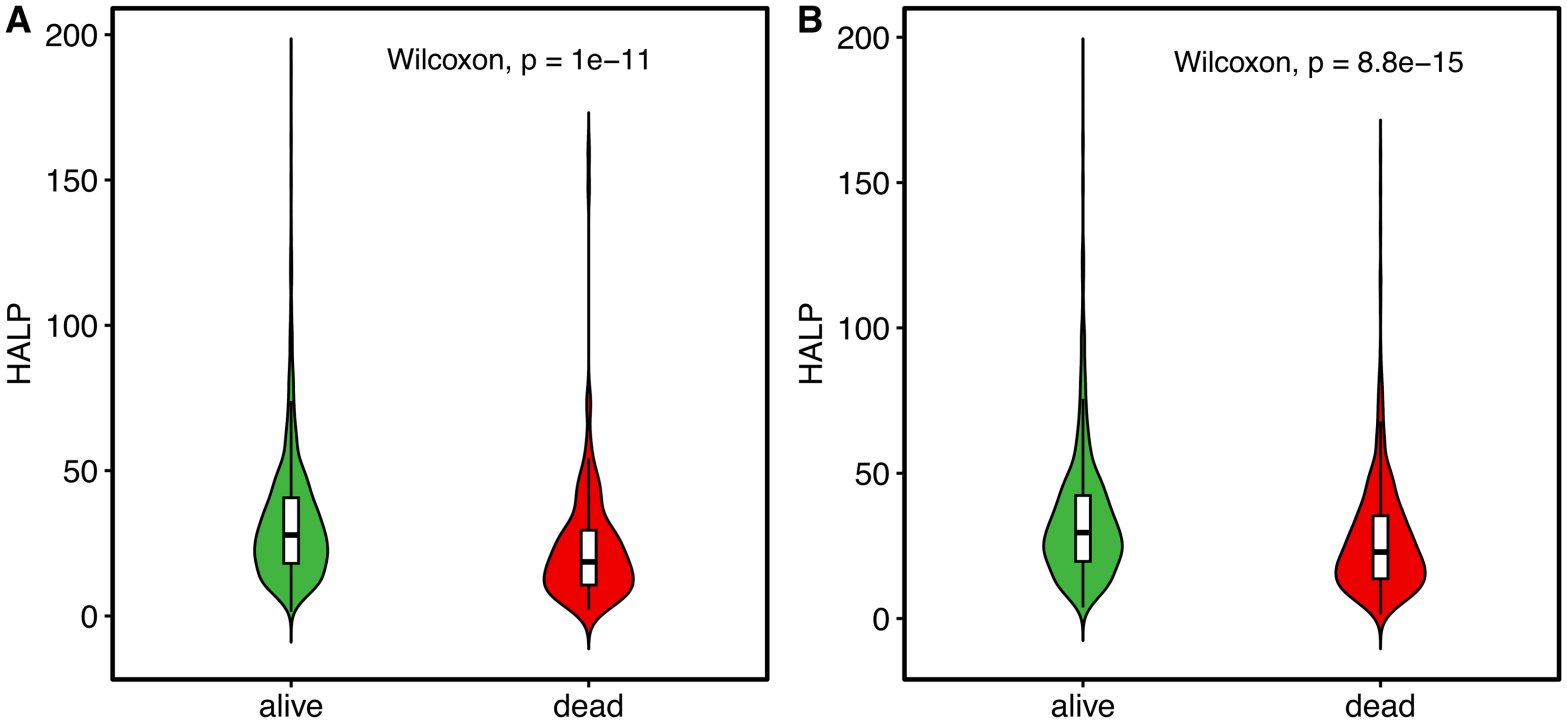
Violin plots of the HALP score between alive (green) and dead (red) patients at the 90-day **(A)** and the longest follow-up **(B)**. The box plots within the violin plots indicate the median and interquartile range. Abbreviation: HALP, hemoglobin, albumin, lymphocyte and platelet.

**Figure 2:**
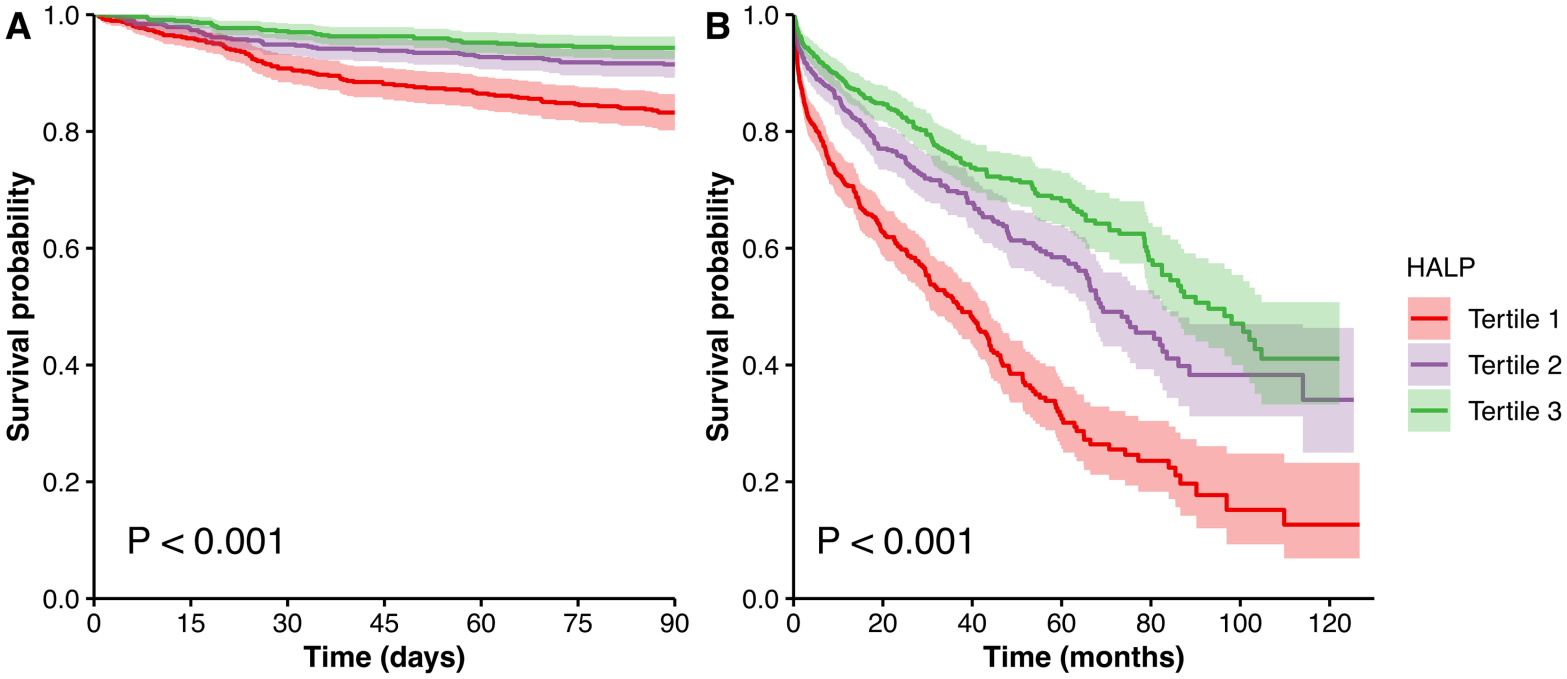
Kaplan–Meier survival curves for survival probability stratified by HALP tertiles at the 90-day **(A)** and the longest follow-up **(B)**. Abbreviation: HALP, hemoglobin, albumin, lymphocyte and platelet.

During a median follow-up period of 44.4 months, 667 patients (39.1%) died, of whom 174 cases (10.2%) died within 90 days. Compared with patients who were alive, the HALP scores were significantly lower in dead patients at the 90-day follow-up [18.6 (10.7, 29.5) vs. 27.8 (18.1, 40.7), *p* < 0.001, Figure 1A], and the longest follow-up [22.9 (13.7, 35.4) vs. 29.6 (19.7, 42.3), *p* < 0.001, Figure 1B]. As presented in Table 2, a higher HALP tertile was correlated with lower 90-day and overall mortality rates (all *p* < 0.001). Similarly, Kaplan–Meier survival analysis revealed significant differences in survival across the HALP tertiles. The lowest survival probability was observed in tertile 1 group at the 90-day follow-up (log-rank *χ^2^* = 40.496, *p* < 0.001, Figure 2A), and the longest follow-up (log-rank *χ^2^* = 120.964, *p* < 0.001, Figure 2B).

**Table 2:** Relationship between the HALP score and mortality risk.

HALP score	90-day mortality					Overall mortality				
	Events, *n* (%)	Unadjusted		Adjusted		Events, *n* (%)	Unadjusted		Adjusted	
		HR (95% *CI*)	*P* value	HR (95% *CI*)	*P* value		HR (95% *CI*)	*P* value	HR (95% *CI*)	*P* value
Tertile 1	94 (16.5)	Reference		Reference		294 (51.7)	Reference		Reference	
Tertile 2	48 (8.4)	0.489 (0.345–0.692)	<0.001	0.676 (0.471–0.972)	0.035	200 (35.1)	0.522 (0.436–0.625)	<0.001	0.681 (0.566–0.820)	<0.001
Tertile 3	32 (5.6)	0.320 (0.214–0.477)	<0.001	0.598 (0.390–0.918)	0.019	173 (30.4)	0.373 (0.308–0.451)	<0.001	0.618 (0.504–0.759)	<0.001
*P* for trend	<0.001		<0.001		0.013	<0.001		<0.001		<0.001
Continuous HALP (per unit)	174 (10.2)	0.972 (0.961–0.982)	<0.001	0.989 (0.979–1.000)	0.044	667 (39.1)	0.977 (0.972–0.982)	<0.001	0.990 (0.985–0.995)	<0.001

HALP, hemoglobin, albumin, lymphocyte and platelet; n, number; HR, hazard ratio; CI, confidence interval. Adjusted for age, sex, body mass index, marital status, smoking, Charlson Comorbidity Index, fracture type, neutrophil, monocyte, creatinine, glucose, international normalized ratio, calcium, sodium and potassium.

After multivariate adjustment, compared to tertile 1, tertile 2 and tertile 3 groups exhibited 0.676-fold (95% *CI*: 0.471–0.972, *p* = 0.035) and 0.598-fold (95% *CI*: 0.390–0.918, *p* = 0.019) lower risks of 90-day mortality, as well as 0.681-fold (95% *CI*: 0.566–0.820, *p* < 0.001) and 0.618-fold (95% *CI*: 0.504–0.759, *p* < 0.001) decreased risks of overall mortality, respectively. When the median value of each HALP tertile was assigned and analyzed as a continuous variable, a statistically significant decreasing trend was observed (*P* for trend <0.05). For each unit increase in the HALP score, there was a significant decrease in 90-day mortality by 1.1%, and in overall mortality by 1.0% (all *p* < 0.05).

**Figure 3:**
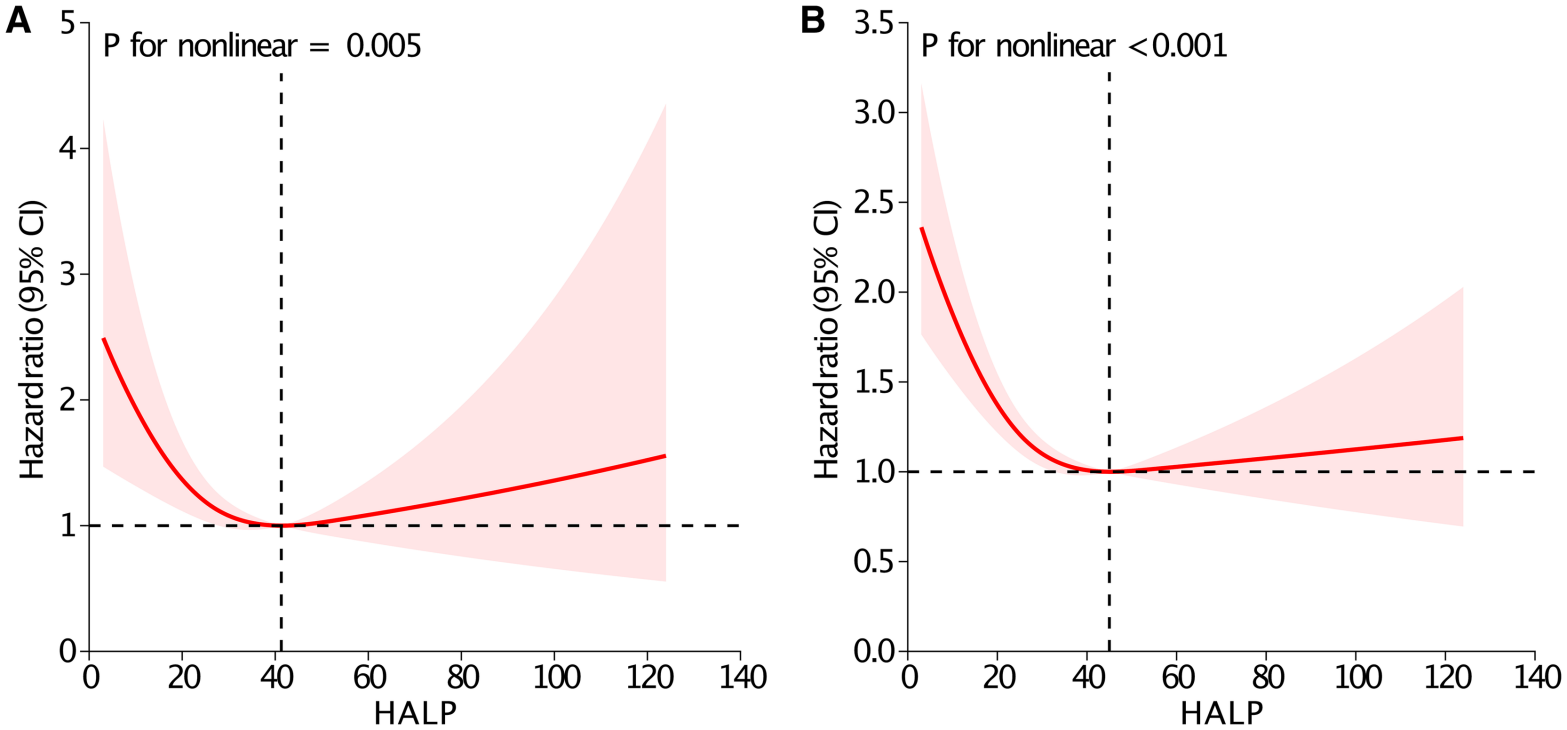
Restricted cubic spline analysis between the HALP score and 90-day **(A)** and overall mortality **(B)**. Adjusted for age, sex, body mass index, marital status, smoking, Charlson Comorbidity Index, fracture type, neutrophil, monocyte, creatinine, glucose, international normalized ratio, calcium, sodium and potassium. Abbreviation: HALP, hemoglobin, albumin, lymphocyte and platelet; CI, confidence interval.

To ensure the reliability of the results, subgroup analyses were conducted across various patient characteristics (Table 3). The findings from the subgroup analyses were in line with the primary results, with no significant interaction effects detected. Additionally, the association remained significant even after excluding patients with missing data (Supplementary Table S4). Furthermore, RCS analyses indicated nonlinear relationships between the HALP score and 90-day mortality (*P* for nonlinear = 0.005, Figure 3A), and overall mortality (*P* for nonlinear <0.001, Figure 3B). The inflection points of the HALP score for 90-day and overall mortality were 41.3 and 45.0, respectively. Below these points, each unit increase in the HALP score corresponded to a significant reduction in mortality risk. Specifically, the risk of 90-day mortality decreased by 2.7% (HR = 0.973, 95% *CI*: 0.955–0.991, *p* = 0.003), and the risk of overall mortality reduced by 2.3% (HR = 0.977, 95% *CI*: 0.969–0.986, *p* < 0.001). However, no significant associations were detected beyond the inflection points for either 90-day mortality (HR = 1.007, 95% *CI*: 0.990–1.024, *p* = 0.437) or overall mortality (HR = 1.005, 95% *CI*: 0.995–1.014, *p* = 0.299).

**Table 3:** Subgroup analysis of the relationship between the HALP score (per unit increase) and mortality risk.

Subgroups	90-day mortality				Overall mortality			
	Events/patients	HR (95% *CI*)	*P* value	*P* for interaction	Events/patients	HR (95% *CI*)	*P* value	*P* for interaction
Age, years				0.345				0.351
<80.0	48/810	0.966 (0.946–0.985)	<0.001		204/810	0.978 (0.970–0.986)	<0.001	
≥80.0	126/897	0.980 (0.967–0.992)	0.001		463/897	0.984 (0.978–0.990)	<0.001	
Sex				0.164				0.572
Male	81/606	0.984 (0.970–0.997)	0.014		268/606	0.980 (0.972–0.987)	<0.001	
Female	93/1101	0.959 (0.942–0.974)	<0.001		399/1101	0.976 (0.969–0.982)	<0.001	
BMI				0.978				0.757
Underweight	36/325	0.976 (0.949–0.999)	0.042		162/325	0.976 (0.963–0.987)	<0.001	
Normal weight	117/1076	0.974 (0.961–0.986)	<0.001		415/1076	0.981 (0.975–0.987)	<0.001	
Overweight	21/306	0.956 (0.923–0.986)	0.002		90/306	0.971 (0.958–0.984)	<0.001	
Marital status				0.720				0.183
Widowed	54/449	0.975 (0.955–0.993)	0.006		208/449	0.983 (0.974–0.992)	<0.001	
Other	120/1258	0.971 (0.958–0.983)	<0.001		459/1258	0.976 (0.970–0.982)	<0.001	
CCI				0.534				0.724
None	34/778	0.984 (0.962–1.002)	0.094		224/778	0.980 (0.971–0.987)	<0.001	
Low	63/519	0.970 (0.951–0.987)	<0.001		236/519	0.979 (0.970–0.987)	<0.001	
High	77/410	0.979 (0.963–0.994)	0.005		207/410	0.984 (0.975–0.993)	<0.001	
Fracture type				0.378				0.124
Femoral neck	69/829	0.978 (0.962–0.992)	0.003		286/829	0.975 (0.967–0.982)	<0.001	
Intertrochanteric	105/878	0.969 (0.953–0.983)	<0.001		381/878	0.983 (0.976–0.989)	<0.001	

HALP, hemoglobin, albumin, lymphocyte and platelet; HR, hazard ratio; CI, confidence interval; BMI, body mass index; CCI, Charlson Comorbidity Index.

### 3.3 Incremental predictive performance of the HALP score

As illustrated in Table 4, adding the HALP score to the CCI model significantly enhanced the predictive ability for all-cause mortality (all *p* < 0.05). Specifically, the c statistic showed a significant increment of 0.073 for 90-day mortality (increasing from 0.517 to 0.590), and an increment of 0.035 for overall mortality (rising from 0.582 to 0.617). Moreover, the continuous NRI values were 19.268 and 15.466% for 90-day and overall mortality, and the IDI values were 2.014 and 2.085% for 90-day and overall mortality, respectively.

**Table 4:** Reclassification and discrimination metrics for predicting mortality risk using the HALP score.

	c statistic		NRI (continuous), %		IDI, %	
	Estimate (95% *CI*)	*P* value	Estimate (95% *CI*)	*P* value	Estimate (95% *CI*)	*P* value
90-day mortality						
CCI model	0.517 (0.434–0.600)		Reference		Reference	
CCI model + HALP score	0.590 (0.488–0.692)	0.029	19.268 (6.985–26.028)	0.012	2.014 (0.369–4.100)	<0.001
Overall mortality						
CCI model	0.582 (0.562–0.602)		Reference		Reference	
CCI model + HALP score	0.617 (0.589–0.645)	<0.001	15.466 (8.836–22.152)	<0.001	2.085 (0.926–3.378)	<0.001

NRI, net reclassification improvement; IDI, integrated discrimination improvement; CI, confidence interval; CCI, Charlson Comorbidity Index; HALP, hemoglobin, albumin, lymphocyte, and platelet.

## 4 Discussion

In our study, higher HALP tertiles were correlated with lower 90-day and overall mortality rates, suggesting an inverse relationship between the HALP score and death risk in older patients with hip fracture. Even after accounting for patient demographics, comorbidity, fracture type and laboratory data, the association remained significant, with a statistically significant decreasing trend across the tertiles. Consistent with our findings, Tian et al. (41) examined this association in patients with acute ischemic stroke, and found that patients in the higher tertiles had lower risks of poor outcomes at both 90 days (HR: 0.61 for tertile 2 and 0.25 for tertile 3, *P* for trend = 0.001), and 1 year (HR: 0.60 for tertile 2 and 0.42 for tertile 3, *P* for trend <0.001) compared to those in the lowest tertile. In the general population, Pan et al. (23) divided 21,578 participants into tertile groups, and also found that participants in tertile 2 and tertile 3 groups had 0.68-fold and 0.80-fold decreased risks of all-cause mortality, as well as 0.60-fold and 0.61-fold decreased risks of cardiovascular mortality (all *P* for trend <0.001). To avoid the influence of cutoff selection on the results, we further analyzed the HALP score as a continuous variable instead of a categorical variable. The analysis also showed that each unit increase in the HALP score corresponded to a 1.1% decrease in the risk of 90-day mortality and a 1.0% decrease in the risk of overall mortality. In addition, similar results were obtained across various subgroup and sensitivity analyses, indicating that the observed relationship was robust. Recently, a systematic review and meta-analysis involving 28 studies with 13,038 patients demonstrated that a low HALP score corresponded to poorer overall survival (HR = 1.61, 95% *CI*: 1.44–1.81). Based on these findings, they concluded that the HALP score was a reliable and negative prognostic indicator for survival outcomes among cancer patients (42).

Moreover, we employed RCS models to assess the potential nonlinear association between the HALP score and mortality. As illustrated in Figure 3, the relationship was clearly nonlinear for both 90-day and overall mortality. To our knowledge, only a few studies have explored whether this association was linear or nonlinear, and these findings have not been entirely consistent. Among patients with acute ischemic stroke, J-shaped nonlinear associations were observed between the HALP score and poor outcomes at 90 days and 1 year (41). Another study showed a nonlinear, U-shaped relationship between the HALP score and both all-cause and cardiovascular mortality in the general population (23). However, no nonlinear relationship was found in hemodialysis patients (*P* for nonlinear = 0.436) (43). In our study, the association between the HALP score and death risk appeared to be U-shaped, which was consistent with the previous study mentioned above (23). Notably, no significant associations were detected beyond the inflection points for either 90-day mortality or overall mortality, suggesting that clinicians should pay more attention to patients with lower HALP score, as they may be at elevated risk for poor survival. Some possible mechanisms might account for the U-shaped relationship. On the one hand, there was a similar U-shaped relationship between hemoglobin and death risk in hip fracture patients (16). Similarly, both low and high levels of hemoglobin have been shown to correlate with an increased risk of mortality in patients with stroke (44), percutaneous coronary intervention (45), and even the general population (46). On the other hand, there was a U-shaped relationship between platelet levels and mortality in emergency department patients (47). Therefore, high or low levels of hemoglobin and platelets may result in this U-shaped association between the HALP score and mortality (23).

Although accumulated evidences support the relationship between a low HALP score and higher mortality, the underlying mechanisms how the HALP score affects the prognosis remain unclear (42). Several potential explanations may account for this association. Firstly, the low hemoglobin level resulting from hip fracture can lead to ischemia and hypoxia, thereby increasing the risk of mortality. Existing studies have established a strong association between anemia and poor survival in patients with hip fracture (16, 17). Secondly, serum albumin is a well-known marker of nutritional status, and its reduction can impair the healing process, exacerbate infections and increase the risk of mortality (17, 18). Thirdly, lymphocyte counts are essential for mediating both adaptive and innate immune responses. A reduction in lymphocyte levels can make individuals more susceptible to viral, bacterial and fungal infections, potentially worsening patient prognosis (18). Lastly, abnormal platelet counts can increase the risk of bleeding and thrombosis, thereby negatively impacting patient outcomes (19).

Given that the CCI model is a well-established tool for predicting mortality in hip fracture patients, we further compared the additional predictive value of the HALP score when incorporated into the CCI model. Our analysis revealed that adding the HALP score significantly improved the predictive ability of CCI model for mortality. This enhancement was evident across various metrics, including c statistic, NRI and IDI, which are crucial indicators of model’s discriminative power. These findings suggest that the HALP score may serve as a useful prognostic indicator for mortality in older patients with hip fracture, potentially leading to more personalized and targeted patient care strategies. At present, only one study has explored the incremental predictive performance of the HALP score, and revealed that incorporating the HALP score improved the prediction of poor outcomes in patients with acute ischemic stroke at both 90 days and 1 year (NRI: 48.38 and 28.95%; IDI: 1.51 and 1.51%; all *p* < 0.05), which was in line with our findings (41). Consequently, the HALP score may be a simple, readily available, and cost-effective prognostic biomarker in older patients with hip fracture.

This study has several strengths. Firstly, consecutive patients were identified from our hip fracture database over a 10-year time period, thus minimizing selection bias. Secondly, the significant association was robust across models, regardless of whether the HALP score was modeled as a continuous or categorical variable, for both 90-day and overall mortality. In addition, subgroup and sensitivity analyses yielded consistent results, suggesting that the association was reliable. Thirdly, this study also explored the incremental predictive value of the HALP score over the CCI model, providing new evidence for possible clinical application.

However, several limitations of this study should be acknowledged. Firstly, this study was retrospective and observational, there may be confounding variables that were not taken into account, including functional status, social support, and established scoring systems for hip fractures [such as the Nottingham Hip Fracture Score (NHFS)]. Due to this reason, we could not compare the additional predictive value of the HALP score over the NHFS model. At the same time, telephone follow-ups are prone to inherent recall bias. To address this issue, we validated study outcomes with medical records, thereby enhancing the accuracy of our results. Secondly, the single-center study design and the relatively modest sample size may restrict the generalizability of the findings. Thirdly, our database did not include some factors known to affect mortality, such as such as hormone therapy, use of anti-osteoporotic medications, and bone metabolism markers. Fourthly, we only obtained the HALP score at admission, and did not evaluate the dynamic change of the HALP score during hospitalization. Therefore, further prospective, large-sample, multicenter studies are needed to validate these findings.

## 5 Conclusion

In summary, the HALP score was independently associated with mortality risk, highlighting its potential as a useful prognostic indicator for predicting mortality in older patients with hip fracture. These findings may assist clinicians in identifying hip fracture patients who are at high risk of mortality and make individualized treatment decisions timely for these patients, thereby improving the prognosis.

## Data Availability

The raw data supporting the conclusions of this article will be made available by the authors, without undue reservation.

## References

[1] Feng JN Zhang CG Li BH Zhan SY Wang SF Song CL. Global burden of hip fracture: the global burden of disease study. Osteoporos Int. (2023) 35:41–52. doi: 10.1007/s00198-023-06907-3, PMID: 37704919

[2] Harris E Clement N MacLullich A Farrow L. The impact of an ageing population on future increases in hip fracture burden. Bone Joint J. (2024) 106-B:62–8. doi: 10.1302/0301-620X.106B1.BJJ-2023-0740.R1, PMID: 38160690

[3] Zhang C Feng J Wang S Gao P Xu L Zhu J. Incidence of and trends in hip fracture among adults in urban China: a nationwide retrospective cohort study. PLoS Med. (2020) 17:e1003180. doi: 10.1371/journal.pmed.1003180, PMID: 32760065 PMC7410202

[4] Sing CW Lin TC Bartholomew S Bell JS Bennett C Beyene K. Global epidemiology of hip fractures: secular trends in incidence rate, post-fracture treatment, and all-cause mortality. J Bone Miner Res. (2023) 38:1064–75. doi: 10.1002/jbmr.4821, PMID: 37118993

[5] Dong Y Zhang Y Song K Kang H Ye D Li F. What was the epidemiology and global burden of disease of hip fractures from 1990 to 2019? Results from and additional analysis of the global burden of disease study 2019. Clin Orthop Relat Res. (2023) 481:1209–20. doi: 10.1097/CORR.0000000000002465, PMID: 36374576 PMC10194687

[6] Zeelenberg ML Den Hartog D Panneman MJM Polinder S Verhofstad MHJ Van Lieshout EMM. Trends in incidence, health care consumption, and costs for proximal femoral fractures in the Netherlands between 2000 and 2019: a nationwide study. Osteoporos Int. (2023) 34:1389–99. doi: 10.1007/s00198-023-06774-y, PMID: 37119329 PMC10382328

[7] GBD 2019 Fracture Collaborators. Global, regional, and national burden of bone fractures in 204 countries and territories, 1990-2019: a systematic analysis from the global burden of disease study 2019. Lancet Healthy Longev. (2021) 2:e580–92. doi: 10.1016/S2666-7568(21)00172-0, PMID: 34723233 PMC8547262

[8] Bui M Nijmeijer WS Hegeman JH Witteveen A Groothuis-Oudshoorn CGM. Systematic review and meta-analysis of preoperative predictors for early mortality following hip fracture surgery. Osteoporos Int. (2023) 35:561–74. doi: 10.1007/s00198-023-06942-0, PMID: 37996546 PMC10957669

[9] McHugh MA Wilson JL Schaffer NE Olsen EC Perdue A Ahn J. Preoperative comorbidities associated with early mortality in hip fracture patients: a multicenter study. J Am Acad Orthop Surg. (2023) 31:81–6. doi: 10.5435/JAAOS-D-21-01055, PMID: 36580049

[10] Lin CQ Jin CA Ivanov D Gonzalez CA Gardner MJ. Using machine-learning to decode postoperative hip mortality trends: actionable insights from an extensive clinical dataset. Injury. (2024) 55:111334. doi: 10.1016/j.injury.2024.111334, PMID: 38266327

[11] Lei M Han Z Wang S Han T Fang S Lin F. A machine learning-based prediction model for in-hospital mortality among critically ill patients with hip fracture: an internal and external validated study. Injury. (2023) 54:636–44. doi: 10.1016/j.injury.2022.11.031, PMID: 36414503

[12] Harris AHS Trickey AW Eddington HS Seib CD Kamal RN Kuo AC. A tool to estimate risk of 30-day mortality and complications after hip fracture surgery: accurate enough for some but not all purposes? A study from the ACS-NSQIP database. Clin Orthop Relat Res. (2022) 480:2335–46. doi: 10.1097/CORR.0000000000002294, PMID: 35901441 PMC10538935

[13] Wu H Li Y Tong L Wang Y Sun Z. Worldwide research tendency and hotspots on hip fracture: a 20-year bibliometric analysis. Arch Osteoporos. (2021) 16:73. doi: 10.1007/s11657-021-00929-2, PMID: 33866438

[14] Schwarz GM Hajdu S Windhager R Willegger M. The top fifty most influential articles on hip fractures. Int Orthop. (2022) 46:2437–53. doi: 10.1007/s00264-022-05511-0, PMID: 35870001 PMC9492587

[15] Peng G Guan Z Hou Y Gao J Rao W Yuan X. Depicting developing trend and core knowledge of hip fracture research: a bibliometric and visualised analysis. J Orthop Surg Res. (2021) 16:174. doi: 10.1186/s13018-021-02292-x, PMID: 33663568 PMC7931604

[16] Zhang BF Wang J Wen PF Wu YJ Guo JB Wang YK. The association between hemoglobin at admission and mortality of older patients with hip fracture: a mean 3-year follow-up cohort study. Eur Geriatr Med. (2023) 14:275–84. doi: 10.1007/s41999-023-00759-0, PMID: 36805525

[17] Pan L Ning T Wu H Liu H Wang H Li X. Prognostic nomogram for risk of mortality after hip fracture surgery in geriatrics. Injury. (2022) 53:1484–9. doi: 10.1016/j.injury.2022.01.029, PMID: 35078620

[18] Blanco JF da Casa C Pablos-Hernández C González-Ramírez A Julián-Enríquez JM Díaz-Álvarez A. 30-day mortality after hip fracture surgery: influence of postoperative factors. PLoS One. (2021) 16:e0246963. doi: 10.1371/journal.pone.0246963, PMID: 33592047 PMC7886122

[19] Asrian G Suri A Rajapakse C. Machine learning-based mortality prediction in hip fracture patients using biomarkers. J Orthop Res. (2024) 42:395–403. doi: 10.1002/jor.25675, PMID: 37727905

[20] Wang Y Jiang Y Luo Y Lin X Song M Li J. Prognostic nutritional index with postoperative complications and 2-year mortality in hip fracture patients: an observational cohort study. Int J Surg. (2023). doi: 10.1097/JS9.0000000000000614, PMID: 37526114 PMC10651254

[21] Wang Z Wang H Yang L Jiang W Chen X Liu Y. High platelet-to-lymphocyte ratio predicts poor survival of elderly patients with hip fracture. Int Orthop. (2021) 45:13–21. doi: 10.1007/s00264-020-04833-1, PMID: 32989560 PMC7521768

[22] Zhao Z Yin XN Wang J Chen X Cai ZL Zhang B. Prognostic significance of hemoglobin, albumin, lymphocyte, platelet in gastrointestinal stromal tumors: a propensity matched retrospective cohort study. World J Gastroenterol. (2022) 28:3476–87. doi: 10.3748/wjg.v28.i27.3476, PMID: 36158264 PMC9346454

[23] Pan H Lin S. Association of hemoglobin, albumin, lymphocyte, and platelet score with risk of cerebrovascular, cardiovascular, and all-cause mortality in the general population: results from the NHANES 1999-2018. Front Endocrinol. (2023) 14:1173399. doi: 10.3389/fendo.2023.1173399, PMID: 37424853 PMC10328756

[24] Zhou J Yang D. Prognostic significance of hemoglobin, albumin, lymphocyte and platelet (HALP) score in hepatocellular carcinoma. J Hepatocell Carcinoma. (2023) 10:821–31. doi: 10.2147/JHC.S411521, PMID: 37288141 PMC10243610

[25] Zhao Z Xu L. Prognostic significance of HALP score and combination of peripheral blood multiple indicators in patients with early breast cancer. Front Oncol. (2023) 13:1253895. doi: 10.3389/fonc.2023.1253895, PMID: 38188308 PMC10768851

[26] Toprak K Toprak İH Acar O Ermiş MF. The predictive value of the HALP score for no-reflow phenomenon and short-term mortality in patients with ST-elevation myocardial infarction. Postgrad Med. (2024) 136:169–79. doi: 10.1080/00325481.2024.2319567, PMID: 38356155

[27] Liu L Gong B Wang W Xu K Wang K Song G. Association between haemoglobin, albumin, lymphocytes, and platelets and mortality in patients with heart failure. ESC Heart Fail. (2024) 11:1051–60. doi: 10.1002/ehf2.14662, PMID: 38243382 PMC10966267

[28] Zheng Y Huang Y Li H. Hemoglobin albumin lymphocyte and platelet score and all-cause mortality in coronary heart disease: a retrospective cohort study of NHANES database. Front Cardiovasc Med. (2023) 10:1241217. doi: 10.3389/fcvm.2023.1241217, PMID: 38028472 PMC10679332

[29] Han H Hu S Du J. Predictive value of the hemoglobin-albumin-lymphocyte-platelet (HALP) index for ICU mortality in patients with acute exacerbations of chronic obstructive pulmonary disease (AECOPD). Intern Emerg Med. (2023) 18:85–96. doi: 10.1007/s11739-022-03132-4, PMID: 36357607

[30] Wang Z Jiang W Chen X Yang L Wang H Liu Y. Systemic immune-inflammation index independently predicts poor survival of older adults with hip fracture: a prospective cohort study. BMC Geriatr. (2021) 21:155. doi: 10.1186/s12877-021-02102-3, PMID: 33663402 PMC7934427

[31] Wang Z Chen X Yang L Wang H Jiang W Liu Y. A new preoperative risk score for predicting mortality of elderly hip fracture patients: an external validation study. Aging Clin Exp Res. (2021) 33:2519–27. doi: 10.1007/s40520-021-01786-2, PMID: 33486721

[32] Chen X Wang Z Liu H Zhang J Zhu Z Chen Y. Muscular calf vein thrombosis is associated with increased 30-day mortality but not 90-day mortality in older patients with hip fracture. Am J Cardiol. (2022) 184:141–6. doi: 10.1016/j.amjcard.2022.08.018, PMID: 36123171

[33] Wang Z Chen X Wu Y Jiang W Yang L Wang H. Admission resting heart rate as an independent predictor of all-cause mortality in elderly patients with hip fracture. Int J Gen Med. (2021) 14:7699–706. doi: 10.2147/IJGM.S333971, PMID: 34764683 PMC8575447

[34] Fang Z Gao B Wang Z Chen X Liu M. Association of systemic inflammation response index with mortality risk in older patients with hip fracture: a 10-year retrospective cohort study. Front Med. (2024) 11:1401443. doi: 10.3389/fmed.2024.1401443, PMID: 38841577 PMC11150681

[35] Çelen ZE. Predictive value of the systemic immune-inflammation index on one-year mortality in geriatric hip fractures. BMC Geriatr. (2024) 24:340. doi: 10.1186/s12877-024-04916-3, PMID: 38622572 PMC11020614

[36] Lu Y Chen W Guo Y Wang Y Wang L Zhang Y. Risk factors for short-term mortality in elderly hip fracture patients with complicated heart failure in the ICU: a MIMIC-IV database analysis using nomogram. J Orthop Surg Res. (2023) 18:829. doi: 10.1186/s13018-023-04258-7, PMID: 37924144 PMC10625197

[37] Li DY Zhang K Wang H Zhuang Y Zhang BF Zhang DL. Preoperative serum calcium level predicts postoperative mortality in older adult patients with hip fracture: a prospective cohort study of 2333 patients. J Am Med Dir Assoc. (2024) 25:655–60. doi: 10.1016/j.jamda.2023.08.004, PMID: 37660723

[38] Li J Guo S Ma R He J Zhang X Rui D. Comparison of the effects of imputation methods for missing data in predictive modelling of cohort study datasets. BMC Med Res Methodol. (2024) 24:41. doi: 10.1186/s12874-024-02173-x, PMID: 38365610 PMC10870437

[39] Kim JH. Multicollinearity and misleading statistical results. Korean J Anesthesiol. (2019) 72:558–69. doi: 10.4097/kja.19087, PMID: 31304696 PMC6900425

[40] Xanthakis V Sullivan LM Vasan RS Benjamin EJ Massaro JM D'Agostino RB Sr. Assessing the incremental predictive performance of novel biomarkers over standard predictors. Stat Med. (2014) 33:2577–84. doi: 10.1002/sim.6165, PMID: 24719270 PMC4047140

[41] Tian M Li Y Wang X Tian X Pei LL Wang X. The hemoglobin, albumin, lymphocyte, and platelet (HALP) score is associated with poor outcome of acute ischemic stroke. Front Neurol. (2020) 11:610318. doi: 10.3389/fneur.2020.610318, PMID: 33510706 PMC7835486

[42] Xu H Zheng X Ai J Yang L. Hemoglobin, albumin, lymphocyte, and platelet (HALP) score and cancer prognosis: a systematic review and meta-analysis of 13,110 patients. Int Immunopharmacol. (2023) 114:109496. doi: 10.1016/j.intimp.2022.109496, PMID: 36462339

[43] Zhang F Li L Shi T Liu Y Xie J Yu L. The hemoglobin, albumin, lymphocyte, and platelet (HALP) is a potent indicator for the prognosis in hemodialysis patients. Medicine (Baltimore). (2023) 102:e33650. doi: 10.1097/MD.0000000000033650, PMID: 37171338 PMC10174384

[44] Zhang R Xu Q Wang A Jiang Y Meng X Zhou M. Hemoglobin concentration and clinical outcomes after acute ischemic stroke or transient ischemic attack. J Am Heart Assoc. (2021) 10:e022547. doi: 10.1161/JAHA.121.022547, PMID: 34845923 PMC9075388

[45] Kim BG Kim H Hong SJ Ahn CM Shin DH Kim JS. Relation of Preprocedural hemoglobin level to outcomes after percutaneous coronary intervention. Am J Cardiol. (2019) 124:1319–26. doi: 10.1016/j.amjcard.2019.07.056, PMID: 31493827

[46] Kawashima M Hisamatsu T Harada A Kadota A Kondo K Okami Y. Relationship between hemoglobin concentration and cardiovascular disease mortality in a 25-year follow-up study of a Japanese general population - NIPPON DATA90. Circ J. (2024) 88:742–50. doi: 10.1253/circj.CJ-23-0725 38382938

[47] Brzeźniakiewicz-Janus K Lancé MD Tukiendorf A Janus T Franków M Rupa-Matysek J. Selected hematological biomarkers to predict acute mortality in emergency department patients. Recent Polish Hospital Statistics. Dis Markers. (2020) 2020:8874361. doi: 10.1155/2020/8874361 32724484 PMC7381964

