# Supplementary material for: The hemoglobin, albumin, lymphocyte, and platelet score as a useful predictor for mortality in older patients with hip fracture
Source: Front Med (Lausanne). 2025 Feb 18;12:1450818. doi: 10.3389/fmed.2025.1450818 (PMC11876120; doi:10.3389/fmed.2025.1450818)
Supplement: Supplementary file 1 [file Table_1.docx]

**Supplementary Table S1.** Univariate Cox regression analyses of factors associated with mortality.

|  | **90-day mortality** | |  | **Overall mortality** | |
| --- | --- | --- | --- | --- | --- |
| **Variables** | **HR (95% *CI*)** | ***P* value** |  | **HR (95% *CI*)** | ***P* value** |
| Age | 1.06 (1.04-1.08) | <0.001 |  | 1.08 (1.07-1.09) | <0.001 |
| Sex (male vs female) | 1.63 (1.21-2.20) | 0.001 |  | 1.33 (1.14-1.55) | <0.001 |
| BMI | 0.96 (0.91-1.00) | 0.048 |  | 0.93 (0.91-0.96) | <0.001 |
| Marital status (widowed vs other) | 1.29 (0.93-1.77) | 0.123 |  | 1.52 (1.29-1.79) | <0.001 |
| Smoking (yes vs no) | 1.48 (1.07-2.05) | 0.016 |  | 1.37 (1.16-1.63) | <0.001 |
| CCI (low vs none) | 2.91 (1.92-4.42) | <0.001 |  | 2.12 (1.77-2.55) | <0.001 |
| CCI(high vs none) | 4.67 (3.12-7.00) | <0.001 |  | 3.37 (2.78-4.09) | <0.001 |
| Fracture type (intertrochanteric vs neck) | 1.47 (1.08-1.99) | 0.014 |  | 1.50 (1.29-1.75) | <0.001 |
| Neutrophil | 1.06 (1.02-1.10) | 0.002 |  | 1.06 (1.04-1.08) | <0.001 |
| Monocyte | 1.72 (1.03-2.86) | 0.037 |  | 1.71 (1.31-2.23) | <0.001 |
| Creatinine | 1.00 (1.00-1.00) | <0.001 |  | 1.00 (1.00-1.00) | <0.001 |
| Glucose | 1.06 (1.03-1.09 | <0.001 |  | 1.06 (1.04-1.08) | <0.001 |
| INR | 8.85 (4.64-16.89) | <0.001 |  | 3.15 (2.01-4.94) | <0.001 |
| Calcium | 0.11 (0.05-0.26) | <0.001 |  | 0.27 (0.17-0.43) | <0.001 |
| Sodium | 0.95 (0.92-0.98) | 0.002 |  | 0.96 (0.94-0.97) | <0.001 |
| Potassium | 1.41 (1.10-1.80) | 0.007 |  | 1.13 (0.98-1.30) | 0.105 |

Abbreviations: HR, hazard ratio; CI, confidence interval; BMI, body mass index; CCI, Charlson Comorbidity Index; INR, international normalized ratio.
