# Supplementary material for: The hemoglobin, albumin, lymphocyte, and platelet score as a useful predictor for mortality in older patients with hip fracture
Source: Front Med (Lausanne). 2025 Feb 18;12:1450818. doi: 10.3389/fmed.2025.1450818 (PMC11876120; doi:10.3389/fmed.2025.1450818)
Supplement: Supplementary file 2 [file Table_2.docx]

**Supplementary Table S2** Multicollinearity analysis with variance inflation factor (VIF) for multivariate Cox analyses.

| **Variables** | **90-day mortality** | | |  | **Overall mortality** | | |
| --- | --- | --- | --- | --- | --- | --- | --- |
| HALP (tertile 2) | 1.553 |  |  |  | 1.553 |  |  |
| HALP (tertile 3) | 1.350 |  |  |  | 1.350 |  |  |
| HALP for trend |  | 1.180 |  |  |  | 1.180 |  |
| Continuous HALP |  |  | 1.179 |  |  |  | 1.179 |
| Age | 1.252 | 1.252 | 1.253 |  | 1.252 | 1.252 | 1.253 |
| Sex | 1.470 | 1.470 | 1.470 |  | 1.470 | 1.470 | 1.470 |
| BMI | 1.127 | 1.124 | 1.118 |  | 1.127 | 1.124 | 1.118 |
| Marital status | 1.182 | 1.182 | 1.182 |  | 1.182 | 1.182 | 1.182 |
| Smoking | 1.353 | 1.351 | 1.352 |  | 1.353 | 1.351 | 1.352 |
| Low CCI | 1.456 | 1.453 | 1.448 |  | 1.456 | 1.453 | 1.448 |
| High CCI | 1.510 | 1.508 | 1.508 |  | 1.510 | 1.508 | 1.508 |
| Fracture type | 1.115 | 1.114 | 1.117 |  | 1.115 | 1.114 | 1.117 |
| Neutrophil | 1.402 | 1.402 | 1.410 |  | 1.402 | 1.402 | 1.410 |
| Monocyte | 1.288 | 1.287 | 1.297 |  | 1.288 | 1.287 | 1.297 |
| Creatinine | 1.255 | 1.253 | 1.254 |  | 1.255 | 1.253 | 1.254 |
| Glucose | 1.275 | 1.272 | 1.272 |  | 1.275 | 1.272 | 1.272 |
| INR | 1.048 | 1.048 | 1.049 |  | 1.048 | 1.048 | 1.049 |
| Calcium | 1.197 | 1.195 | 1.192 |  | 1.197 | 1.195 | 1.192 |
| Sodium | 1.084 | 1.084 | 1.083 |  | 1.084 | 1.084 | 1.083 |
| Potassium | 1.112 | 1.111 | 1.111 |  | 1.112 | 1.111 | 1.111 |

Abbreviations: HALP, hemoglobin, albumin, lymphocyte and platelet; BMI, body mass index; CCI, Charlson Comorbidity Index; INR, international normalized ratio.
