# Supplementary material for: The hemoglobin, albumin, lymphocyte, and platelet score as a useful predictor for mortality in older patients with hip fracture
Source: Front Med (Lausanne). 2025 Feb 18;12:1450818. doi: 10.3389/fmed.2025.1450818 (PMC11876120; doi:10.3389/fmed.2025.1450818)
Supplement: Supplementary file 3 [file Table_3.docx]

**Supplementary Table S3** Akaike information criteria values of the HALP score explored with restricted cubic spline.

|  | **Knots = 3** | **Knots = 4** | **Knots = 5** | **Knots = 6** | **Knots = 7** | **Optimal number of knots** |
| --- | --- | --- | --- | --- | --- | --- |
| 90-day mortality | 2408.2 | 2409.9 | 2411.8 | 2413.4 | 2415.3 | 3 |
| Overall mortality | 8502.9 | 8504.7 | 8506.7 | 8508.1 | 8509.2 | 3 |

Adjusted for age, sex, body mass index, marital status, smoking, Charlson Comorbidity Index, fracture type, neutrophil, monocyte, creatinine, glucose, international normalized ratio, calcium, sodium and potassium.
