# Supplementary material for: The hemoglobin, albumin, lymphocyte, and platelet score as a useful predictor for mortality in older patients with hip fracture
Source: Front Med (Lausanne). 2025 Feb 18;12:1450818. doi: 10.3389/fmed.2025.1450818 (PMC11876120; doi:10.3389/fmed.2025.1450818)
Supplement: Supplementary file 4 [file Table_4.docx]

**Supplementary Table S4** Association between the HALP score and mortality in the complete data (*n* = 1440).

|  | **90-day mortality** | | | | |  | **Overall mortality** | | | | |
| --- | --- | --- | --- | --- | --- | --- | --- | --- | --- | --- | --- |
| **HALP score** | **Events, *n* (%)** | **Unadjusted** | | **Adjusted** | |  | **Events, *n* (%)** | **Unadjusted** | | **Adjusted** | |
|  |  | **HR (95% *CI*)** | ***P* value** | **HR (95% *CI*)** | ***P* value** |  |  | **HR (95% *CI*)** | ***P* value** | **HR (95% *CI*)** | ***P* value** |
| Tertile 1 (*n*=473) | 66 (14.0) | Reference |  | Reference |  |  | 227 (48.0) | Reference |  | Reference |  |
| Tertile 2 (*n*=489) | 30 (6.1) | 0.422 (0.274-0.649) | <0.001 | 0.572 (0.362-0.902) | 0.016 |  | 146 (29.9) | 0.488 (0.396-0.602) | <0.001 | 0.611 (0.492-0.758) | <0.001 |
| Tertile 3 (*n*=478) | 19 (4.0) | 0.270 (0.162-0.449) | <0.001 | 0.510 (0.297-0.875) | 0.015 |  | 121 (25.3) | 0.342 (0.273-0.428) | <0.001 | 0.567 (0.448-0.719) | <0.001 |
| *P* for trend | <0.001 |  | <0.001 |  | 0.009 |  | <0.001 |  | <0.001 |  | <0.001 |
| Continuous HALP (per unit) | 115 (8.0) | 0.961 (0.947-0.975) | <0.001 | 0.981 (0.967-0.995) | 0.009 |  | 494 (34.3) | 0.973 (0.967-0.979) | <0.001 | 0.986 (0.980-0.992) | <0.001 |

Abbreviations: HALP, hemoglobin, albumin, lymphocyte and platelet; n, number; HR, hazard ratio; CI, confidence interval.

Adjusted for age, sex, body mass index, marital status, smoking, Charlson Comorbidity Index, fracture type, neutrophil, monocyte, creatinine, glucose, international normalized ratio, calcium, sodium and potassium.
