# Supplementary figures and images for: The hemoglobin, albumin, lymphocyte, and platelet score as a useful predictor for mortality in older patients with hip fracture
Source: Front Med (Lausanne). 2025 Feb 18;12:1450818. doi: 10.3389/fmed.2025.1450818 (PMC11876120; doi:10.3389/fmed.2025.1450818)

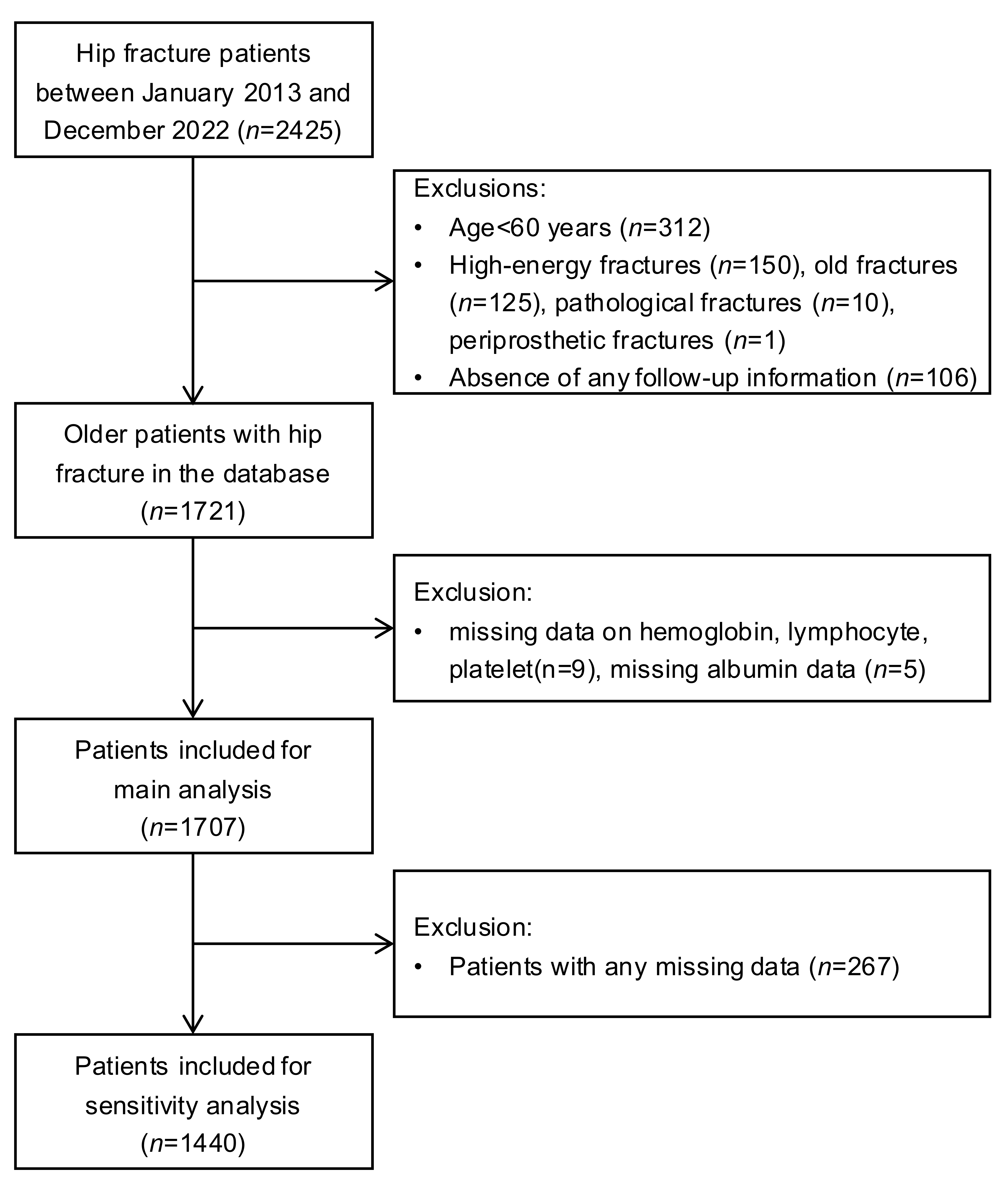

Supplement: Supplementary file 5 [file Image_1.jpeg]
